# Data on recent measles cases at the Jalal-Abad city of Kyrgyzstan

**DOI:** 10.6026/973206300200901

**Published:** 2024-08-31

**Authors:** Temirov Nemat Moidunovich, Temirova Vazira Nematovna, Zholdoshev Saparbai Tezekbayevich, Mamaev Tugolbai Mamaevich, Arstanbekov Sabyrbek Rustamovich

**Affiliations:** 1 Jalal-Abad State University, Medical Faculty, Jalal-Abad, Kyrgyzstan; 2 Kyrgyz Scientific Center for Human Reproduction, Bishkek, Kyrgyzstan; 3 Department of Epidemiology, Microbiology and Infectious Diseases, Osh State University, Osh City, Kyrgyzstan; 4 Department of Public Health, Medical Faculty, Osh State University, Osh City, Kyrgyzstan; 5 Department of Orthopedic Dentistry, Medical Faculty, Osh State University, Osh City, Kyrgyzstan

**Keywords:** Measles, Preventive vaccinations, Kyrgyz, mumps and rubella (MMR), rubella-measles vaccine

## Abstract

Measles caused by the virus is highly infectious and sometimes fatal, but it is prevented through vaccination. In Kyrgyzstan, measles cases were initially documented in September 2023, with a notable escalation observed between November and December of the same year. Children aged 3-5 years and 1-2 years exhibited the highest prevalence rates, followed by infants under one year old. Unvaccinated children constituted the majority of cases, with schoolchildren and kindergarten attendees also affected. Surprisingly, a significant proportion of measles cases occurred in vaccinated children, primarily those who received the MMR or Rubella-measles vaccine. Doctors identified 22 recent measles outbreaks and none of the 75 contacts recorded in these outbreaks acquired the disease. Therefore, it is of interest to report an analysis of measles outbreak spike at Jalal-Abad city of Kyrgyzstan in 2023.

## Background:

Measles infection is a highly contagious acute viral disease that is transmitted by airborne droplets and can lead to serious complications (croup, pneumonia) and sometimes death [1]. Despite the preventive measures aimed at eliminating measles carried out in all countries of the world, this problem remains relevant [2]. Measles cases have been reported in many European countries, Southeast Asian countries, parts of Africa, the Russian Federation, the Republic of Kazakhstan, and the Kyrgyz Republic [3^3^]. In order to stop the spread of measles, vaccination is the best option. Getting the vaccination helps the body fight against the infection and is safe. [4]. A significant percentage of the population that has not been vaccinated and has never had measles, in addition to inadequate vaccination coverage in indicator groups of children and occupational risk groups of adults are factors that indicate the complexity of the epidemic situation. [5]. The World Health Organization reports that in the Russian Federation, the greatest incidence rates are seen in children less than one year old, and that the age range of 25-39 accounts for the biggest portion of the morbidity structure. [6]. Therefore, it is of interest to establish the patterns in the spread of measles infection so as to evaluate the effectiveness of improving epidemiological surveillance of them.

## Methodology:

**Figure 1:**
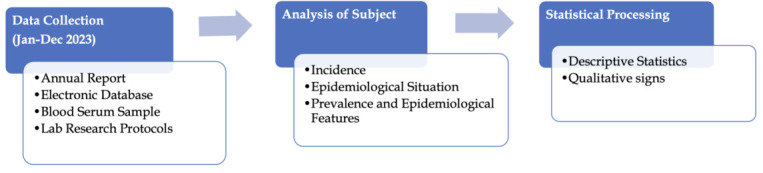
Flowchart showing the methodology for collecting and analyzing data on patients with confirmed measles infection from Jan 2023 to Dec 2023.

The data of patients with confirmed measles infection from January 2023 to December 2023 were collected from the Annual reports of the Department of Disease Prevention and State Sanitary and Epidemiological Supervision of the Ministry of Health of the Kyrgyz Republic using Form No. 1 and 18 to store in databases. The analysis of the incidence of measles infection is done using annual dynamics, age group and gender. Assessment of epidemiological features for measles infection is then completed. Data was subjected to statistical processing using the Excel software package as shown in Figure 1.

## Results and Discussion:

The measles and rubella elimination program has been implemented in accordance with the directive documents of the Ministry of Health of the Kyrgyz Republic and with the WHO strategy for 20 years at the Jalal-Abad city of Kyrgyzstan. Immunization of children against measles is carried out according to the scheme: the first vaccination at the age of 12 months, the second - at 6 years old [7]. Further, "cleaning up" campaigns are regularly conducted. Table 1 show that the measles incidence rate was observed as 4.2 per 1000 children (in the territory of the group of family doctors No. 8) and 3.8 per 1000 children (center for family medicine of the city). However, the onset of measles infection was 0.007 per 1000 children in the city during April [8]. A group of family doctors reported the first case of measles in early September. A child under one year old fell ill and two cases simultaneously in one family who were students of secondary school No. 1, 3rd-6th grade. This is 0.6 per 1000 children compared to 0.1 per 1000 at the city's family medicine center. In October, four measles patients, two secondary school No. 7 students, and two disorganized kids were recorded. This is 0.7 per 1000 children vs 1.4 per 1000 according to the city's Central Medical Committee. The group of family physicians' epidemiological data got bad since October with 1.2 cases per 1000 children reported in October and 1.7 in December (compared to 1.5 and 0.6 instances according to the city's CSM). Nevertheless, morbidity increased in October (1.4 per 1000 children) as per the city's family medical center.

The city's family medical center had 9.98 measles cases per 1,000 children under 14, compared to 10.7 per 1,000. Children aged 3-5 and 1-2 years old had the highest prevalence rate (17.7 and 17.5 per 1000 children respectively) while children under one year old had the second highest (11.2 per 1000 children). The city's family medical facilities found that more children under one year old became sick initially, followed by 1-2 and 3-5 years old, as indicated in Table 2. Within the Group of Family Doctors, 3.1 per 1000 children (compared to -0.4 per 1000 in city family medical clinics). However, there were no instances among adolescents and among adults. According to Table 3, the family doctor group's patients were mostly disorganized children (54.5%), followed by students (22.7%) and kindergarteners (13.6%). Adult housewives and non-workers were sick 4.6% each. The city's family medical center reported measles in 2.5% of unemployed individuals, 1.7% of housewives who cared for sick children, a kindergarten worker, and 0.4% of school instructors.

Among children with measles in the group of family doctors No. 8, 31.8% of children (against 14.5% according to the center for family medicine of the city) received preventive measles vaccination, including the first vaccination against measles, mumps and rubella - 71.4% (according to the center for family medicine of the city - 76.8%) and the second vaccination of rubella measles vaccine 28.6% (according to the center of family doctors of the city - 23.2%) [5]. Unvaccinated children fell ill (59.1%), including by age (7.7%), by medical withdrawal (15.3%) and due to the refusal of parents from preventive vaccinations (77%). Half of the refusal did it for religious reasons. Others doubted the quality and safety of the vaccine. The annual increase in refuse of preventive vaccinations is not only in the territory of the group of family doctors, but also in general at the center of family medicine of the city and in the region. For 9.1% of children, there is no data on receiving a preventive vaccination against measles; there is no form No. 63 or visitors as shown in Table 4 [9]. There were 75 contacts in the group of family physicians' 22 epidemiological foci of measles infection, but no cases (Table 5). Family medical centers are health touchpoints in the city where 5.3% of youngsters were sick. A group of family physicians found focal morbidity in one home and at school, with one case in 14 foci and 2 cases in 4 children. Data further shows that 75 contact persons have received measles immunizations to avoid the disease.

## Conclusions:

Rising infection rates among children aged 1-5 underline the need for personalized care based on demographic data. Data shows that 54.5% of measles patients were young children. This emphasizes the critical need of early vaccinations and targeted education to reduce vaccine resistance. Immunizations serve to reduce the incidence of measles infections. However, significant decreases in vaccination rates resulting from physician withdrawal and parental rejection need thorough public health efforts aimed at 100% vaccination coverage and eliminating of vaccine misconceptions.

## Data availability:

The raw data supporting the conclusions of this article will be made available by the authors, without undue reservation.

## Funding Statement:

The authors received no financial support for the research, authorship, or publication of this article

## Figures and Tables

**Table 1:** The incidence of measles in the population by month in the territory of the family doctors group No. 8 and in the family medicine centers of the city of Jalal-Abad (per 1000 children).

**Months**	**IV**	**V**	**VI**	**VII**	**VIII**	**IX**	**X**	**XI**	**XII**	**Total**
Family Doctors Group N8	-	-	-	-	-	3	4	6	9	22
	-	-	-	-	-	0,6	0,7	1,2	1,7	4,2
Family Medicine Center in Jalal-Abad	1	8	2	-	5	13	178	186	82	475
	0,007	0,06	0,01	-	0,03	0,1	1,4	1,5	0,6	3,8

**Table 2:** Incidence of measles of the population by age categories in the territory of the group of family doctors No. 8 and in the family medicine center of the city (per 1000 children) of Jalal-Abad

	**Age in years**								**Total**
Family Doctors Group No 8	0-1	05-Feb	09-Jun	14-Oct	15-17	18-19	20-29	>30 Y	
	1	13	4	2	-	-	2		22
	11.2	53,2	6.7	2.9	-	-	3,1		4.2
Family Medicine Center	78	251	82	38	5	-	12	9	475
	23,8	33,7	7,0	2,7	0,7	-	0,5	0,2	3,8

**Table 3:** Distribution of measles cases among different patient groups

**Family Doctors Group No 8**	**Indicator**	**Disorganized children**	**Kindergarten**	**School children**	**Non-working**	**Employees of preschool institutions and schools**	**Total**
	Absolute number	12	3	5	2	-	22
	specific gravity	54,5	13,6	22,7	9,2	-	100
Family Medicine Center	Absolute number	296	45	112	20	2	475
	specific gravity	62,3	9,5	23,6	13,4	0,4	100

**Table 4:** Information on the incidence of measles among the population who received preventive vaccinations and were not vaccinated against measles infection in the group of family doctors No. 8 and in the center of family medicine of the city

**Family Doctors Group No 8**	**Indicator**	**Vaccinated**	**Not-Vaccinated**	**By age**	**medical taps**	**Refusal of the vaccine**	**Unknown**	**Total**
	Absolute number	7	13	1	2	10	2	22
	specific gravity	31,8	59,1	7,7	15,3	77,0	9,1	100
Family Medicine Center	Absolute number	69	304	79	19	206	102	475
	specific gravity	14,5	64,0	26,0	6,2	67,8	21,5	100

**Table 5:** Distribution of the focal incidence of measles infection in the group of family doctors No. 8 and in the center of family medicine in the city of Jalal-Abad

**Family Doctors Group No. 8**	**Total cases**	**Total contact details**	**Some of them got sick**		**Foci with the case**			
			Absolute number	Specific gravity	1	2	3	4
	22	75	-	-	14	4		
Family Medicine Center	475	1914	102	5,3	359	45	6	2
